# Comparative *in vitro* susceptibility of clinical *Leishmania* isolates to miltefosine and oleylphosphocholine

**DOI:** 10.3389/fphar.2025.1688856

**Published:** 2025-10-27

**Authors:** Ali Khamesipour, Minoo Tasbihi, Akram Mir Amin Mohammadi, Jodie Dixon, David J. Clark, Henry M. Staines, Sanjeev Krishna, Vanessa Yardley, Simon L. Croft, Neal Alexander, Katrien Van Bocxlaer

**Affiliations:** ^1^ Center for Research and Training in Skin Diseases and Leprosy, Tehran University of Medical Sciences, Tehran, Iran; ^2^ York Biomedical Research Institute, Skin Research Centre, Hull York Medical School, University of York, York, United Kingdom; ^3^ Institute for Infection and Immunity, City St George’s University of London, London, United Kingdom; ^4^ St George’s University Hospitals NHS Foundation Trust, London, United Kingdom; ^5^ Institut für Tropenmedizin, Universitätsklinikum Tübingen, Tübingen, Germany; ^6^ Centre de Recherches Médicales de Lambaréné, Lambaréné, Gabon; ^7^ Department of Infectious and Tropical Diseases, London School of Hygiene and Tropical Medicine, London, United Kingdom; ^8^ Department of Infectious Disease Epidemiology, London School of Hygiene and Tropical Medicine, London, United Kingdom

**Keywords:** cutaneous leishmaniasis, oleylphosphocholine, clinical isolates, dose response, miltefosine

## Abstract

Cutaneous leishmaniasis (CL) is a neglected tropical disease caused by protozoan parasites of the genus *Leishmania*. It poses a significant global health burden, particularly because treatment options are limited. More effective and safer treatments are urgently needed. In previous studies, oleylphosphocholine (OlPC), a novel investigational compound structurally related to miltefosine, exhibited comparable activity to miltefosine in intramacrophage assays across various CL-causing laboratory strains and demonstrated superior efficacy in an experimental CL model. This study investigated the *in vitro* activity of OlPC against clinical isolates of *Leishmania* spp., comparing its activity with standard anti-leishmanial drugs, including miltefosine, amphotericin B, and pentavalent antimonial agents. Seventy *ex vivo* isolates (*L. major* and *L. tropica*) obtained directly from CL patients before any treatment were used to capture the diversity of drug susceptibilities in circulating parasite populations. Dose-response curves were fitted using a four-parameter log-logistic model to estimate EC_50_ and EC_90_ values. Additionally, a linear mixed-effects model was applied to examine the influence of drug type and species on EC_50_ values while accounting for within-isolate variability. Our findings indicate that OlPC exhibits potent *in vitro* anti-leishmanial activity, exceeding that of miltefosine in our *in vitro* intramacrophage model. To facilitate similar analyses, we provide a dedicated wrapper function in R designed to simplify curve fitting and parameter estimation, making the process more accessible to researchers.

## Introduction

Cutaneous leishmaniasis (CL) is a significant global health challenge, affecting millions worldwide, particularly in resource-limited tropical and subtropical regions (Alvar et al., 2012). The disease is characterized by skin lesion(s), which may range from simple small, self-healing to larger, more persistent ulcers (de Vries and Schallig, 2022). These lesions can cause significant scarring, long-term morbidity, and psychosocial distress for affected individuals (Bennis et al., 2018; Nuwangi et al., 2024). The treatment choice is influenced by several factors, including the infectious agent (species of parasite), lesion characteristics (number, size, extent, and location), and treatment availability (Mohammed et al., 2023). Although several treatment options for CL exist, including pentavalent antimonials, amphotericin B, and miltefosine, they are often hampered by significant limitations such as (i) systemic toxicity, requiring careful monitoring; (ii) lengthy treatment courses, often involving multiple injections or extended periods of oral medication that contribute to poor treatment adherence; and (iii) variable efficacy, depending on the *Leishmania* species involved, the patient’s immune status, and other factors (Mowbray et al., 2018). These combined challenges underscore the urgent and unmet need for novel, safer, more effective, and ideally shorter-duration therapies for cutaneous leishmaniasis (Alvar et al., 2018).

Miltefosine, an alkylphosphocholine, has become an important first-line oral treatment for CL, offering a more convenient administration route than injectable therapies (Dorlo et al., 2012). Miltefosine exerts its anti-leishmanial activity through multiple mechanisms targeting parasite membrane integrity and intracellular signaling (Dorlo et al., 2012; Benaim and Paniz-Mondolfi, 2024). Being an alkylphosphocholine, the drug integrates into the parasite’s cell membrane, disrupting its structure and normal lipid metabolism, which impairs parasite viability. Beyond this, miltefosine interferes with essential internal processes, including mitochondrial function and phospholipid production, ultimately leading to parasite death. Although these effects are well-documented, the precise molecular targets can vary between species (Pinto-Martinez et al., 2018), which partly explains the observed differences in treatment efficacy (Van Bocxlaer et al., 2023).

Despite its widespread use, the cure rates for miltefosine treatment differ depending on the *Leishmania* species responsible for infection, geographical location, and host immune response, posing a challenge to achieving consistent treatment outcomes (Monge-Maillo and López-Vélez, 2015; Soto and Berman, 2006). Miltefosine has a long half-life, approximately 150 h–200 h, which contributes to its prolonged therapeutic effects but also raises concerns regarding resistance development. Teratogenicity limits its use in pregnant women, and its common adverse events include nausea and gastrointestinal disturbances, which negatively impact patient compliance (Uranw et al., 2013).

Oleylphosphocholine (OlPC) was developed as a synthetic analog of miltefosine, and it was identified through the exploration of the class of alkylphosphocholines known for their anti-tumor (Unger et al., 1992) and anti-parasitic properties (Croft et al., 1996; Escobar et al., 2001). OlPC was evaluated for its potential to retain oral bioavailability and anti-leishmanial potency while offering an improved safety and pharmacokinetic profile. *In vitro*, OlPC demonstrated similar activity to miltefosine in an intracellular macrophage model using a laboratory-adapted panel of CL-causing species (Van Bocxlaer et al., 2023). In experimental CL models, oral administration of OlPC showed superior efficacy to miltefosine in several studies (Van Bocxlaer et al., 2023; Fortin et al., 2014). More recently, a study suggested that OlPC outperformed miltefosine in canine leishmaniasis, although the treatments were administered at different dosages (4 mg/kg/day for OlPC vs. 2 mg/kg/day for miltefosine, which are equally the respective NOAELs (no observed adverse effect levels) for each drug, with the primary endpoint focusing on clinical signs (Lima et al., 2025). Building on these promising preclinical findings, in this study, we report the susceptibility of 70 *ex vivo* clinical *L. major* and *L. tropica* isolates to miltefosine, OlPC, meglumine antimoniate (pentavalent antimony, SbV), and amphotericin B using an intramacrophage assay. To quantify drug potency, dose-response curves were generated and analyzed using a four-parameter log-logistic regression model, estimating EC_50_ and EC_90_ values, along with Hill slopes and R^2^ values in R. Given the large dataset, a custom wrapper function (Supplementary Material S2) was developed to automate curve generation and fitting, thus ensuring efficient and reproducible analysis using open-source software.

## Materials and methods

### Drugs and formulations

OlPC (Mw 433.61 g/mol) and miltefosine (Mw 407.6 g/mol) were donated by Oblita Therapeutics (Zoersel, Belgium) and Paladin Labs Inc. (Montréal, Canada), respectively. Stock solutions (20 mM) of both compounds were prepared in phosphate-buffered saline (PBS, 0.9% (w/v) NaOH, pH 7.4; Sigma-Aldrich, United Kingdom) and were filter-sterilized and stored at −20 °C until use. Amphotericin B deoxycholate (Fungizone, E.R. Squibb & Sons, United Kingdom) was commercially obtained in 50 mg of amphotericin B (Mw 924.1 g/mol) and prepared according to the manufacturer’s guidelines, resulting in a stock concentration of 5 μg/mL. Meglumine antimoniate powder (containing 55.5% pentavalent antimony (Sb^V^); batch number: 102989254) was solubilized in water, filter-sterilized, and stored at −20 °C until use. Podophyllotoxin was purchased from Sigma-Aldrich (Gillingham, United Kingdom), and stock solutions of 1 mM were prepared in DMSO and stored at −20 °C until use. AlamarBlue was obtained from Thermo Fisher Scientific (Loughborough, United Kingdom).

### Clinical isolates

After obtaining written consent, the skin of patients suspected of being infected with *Leishmania* was wiped with 70% ethanol, and a smear sample was collected from the margins of the lesion using a sterile surgical scalpel. The smear sample was divided into two parts. The first part was smeared onto a microscope slide, fixed with methanol, stained with Giemsa, and examined under a light microscope for the detection of amastigotes. The second part was used to inoculate Novy–MacNeal–Nicolle (NNN) medium overlaid with RPMI-1640 medium (Gibco, Invitrogen, Carlsbad, CA, United States) that was subsequently incubated at 26 °C ± 1 °C. The liquid phase was examined under a light microscope every other day to observe motile promastigotes. When present, parasites were subcultured in RPMI-1640 medium supplemented with 10% (v/v) fetal calf serum (FCS). A small aliquot was removed for DNA extraction and PCR identification, as described previously (Hosseini et al., 2020). All clinical isolates were maintained as promastigotes and used for drug susceptibility testing at or before passage 4.

### Intracellular amastigote drug susceptibility evaluation

Female BALB/c mice (7–10 weeks old) received an intraperitoneal injection with a 2% (w/v) starch suspension (Aq). Twenty-four hours later, peritoneal macrophages (PEMs) were collected by abdominal lavage with RPMI-1640. Subsequently, they were washed, counted, and re-suspended in the RPMI-1640 medium supplemented with 10% (v/v) FCS. Aliquots containing 4 × 10^4^ PEMs were transferred to 16-well Lab-Tek slides (Thermo Fisher Scientific) and incubated overnight at 37 °C in an atmosphere of 5% CO_2_ in air. The next day, stationary-phase *L. major* and *L. tropica* promastigotes, resuspended in RPMI-1640 with 10% (v/v) FCS medium, were added to the macrophages in a 3:1 and 5:1 ratio, respectively.

Twenty-four hours after infection, the overlay and any non-adherent macrophages and promastigotes were removed. Miltefosine and OlPC solutions (40, 5, 0.5, and 0.1 μg/mL final in-well concentrations) were applied to the infected macrophages and incubated for 72 h at 34 °C and 5% CO_2_. Each experiment included a 72-h control (untreated infected control) and Fungizone (amphotericin B, included over a concentration range of 5, 1, 0.25, and 0.01 μg/mL) and a pentavalent antimonial control, which was included over a concentration range of 20, 10, 8, 2, 0.5, and 0.1 µg meglumine antimoniate/mL (equivalent to 11.1, 5.6, 4.4, 1.1, 0.28, and 0.06 µg Sb^V^/mL, respectively). The amastigote burden was determined by microscopic evaluation (x 100 magnification) and compared with the untreated 72-h control.

### Cytotoxicity evaluation

Cytotoxicity was assessed using differentiated THP-1 cells and primary human dermal fibroblasts (NHDF, passage ≤8). THP-1 cells were stimulated with 20 ng/mL PMA for 3 days prior to drug exposure, while NHDF were maintained in DMEM supplemented with 10% (v/v) FCS and non-essential amino acids. A total of 20,000 cells were seeded in 96-well plates and exposed to serial 1:3 dilutions of OlPC or miltefosine, starting at 300 µM. Podophyllotoxin was included as a positive control (starting concentration 0.3 µM). Untreated controls and blanks (medium only) were included, and each compound was tested in triplicate. After 72 h incubation at 37 °C with 5% CO_2_, cell viability was assessed using AlamarBlue^®^ (20 µL/well), incubated for 2 h–4 h, and read at EX/EM 560/585 nm (cut-off at 570 nm) on a CLARIOstar plate reader. EC_50_ values were calculated using nonlinear sigmoidal curve fitting (variable slope) in GraphPad Prism. The selectivity index was calculated as follows:

$$SI=\frac{{CC}_{50}\;\left(host\;cells\right)}{{EC}_{50}\;\left(Leishmania\right)}.$$

### Data processing and analysis

Dose-response models were fitted using a four-parameter log-logistic regression model. In the drc R package, this is undertaken with the drm and LL.4 functions. For each drug, separate models were fitted for each *Leishmania* isolate, enabling the estimation of EC_50_ (the concentration at which 50% inhibition was observed) and EC_90_ values, along with Hill slopes and R^2^ values, on a within-subject basis. Summary statistics (the mean, standard deviation, minimum, and maximum inhibition) were then calculated across the 70 *Leishmania* isolates for each combination of drug concentration and species. Consequently, estimates such as the mean and 95% confidence interval of EC_50_ reflect between-person variation. Inhibition curves for each drug were plotted using ggplot2, with concentration represented on a logarithmic scale, and species-specific differences were visualized.

To compare EC_50_ values between (i) OlPC and miltefosine and between (ii) *L. major* and *L. tropica*, a mixed-effects analysis of variance model was used, with person being a random effect (using the lmer function of the lme4 R package). Drug comparisons are within-subject (same person), whereas species comparisons are between-subject (different people).

The analysis was carried out using R (version 4.2.1 or higher). In addition to those already mentioned, the packages dplyr, data.table, and lmerTest were used.

## Results

### *In vitro* anti-leishmanial activity

**FIGURE 1:**
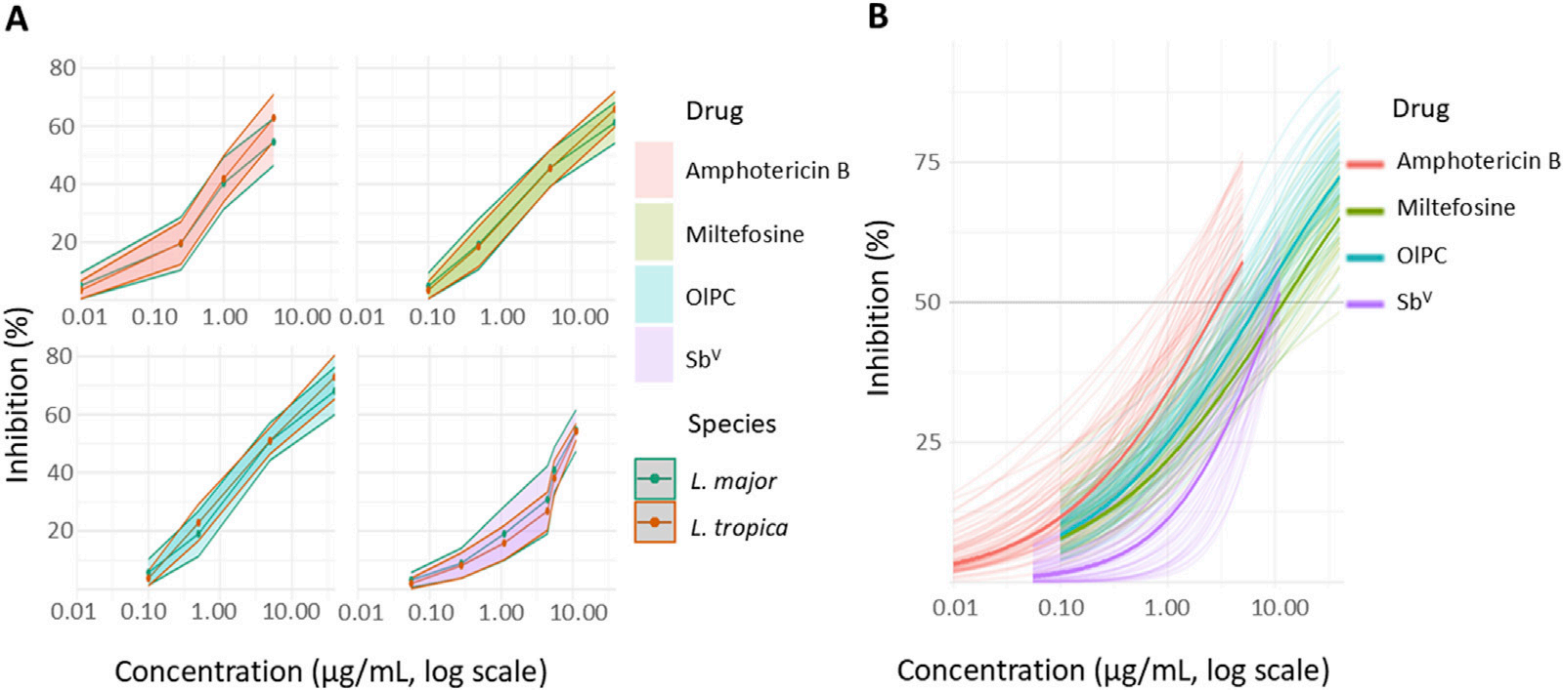
**(A)** Dose-response curves of amphotericin B, pentavalent antimonials (meglumine antimoniate), miltefosine, and OlPC against 34 *L. major* and 36 *L. tropica* clinical isolates. **(B)** Four-parameter logistic model prediction.

In terms of EC_50_ values, the anti-leishmanial activity of the four drugs when ranked from the least to the highest activity is miltefosine < Sb^V^ < OlPC < amphotericin B (Table 1; Supplementary Table S1 for EC_50_ and EC_90_ in µg/mL and µM, respectively). The Hill slope, which describes the steepness of the dose-response curve, was less than 1 for all drugs, indicating a shallow dose-response curve, characteristic of a gradual transition between partial and full inhibition (Motulsky and Christopoulos, 2010) (Figure 1). Yet, it is the highest for meglumine antimoniate, followed by amphotericin B and then OlPC and miltefosine. The goodness-of-fit for dose-response models was high across all drugs, with *R* ^2^ above 94%.

**TABLE 1:** Susceptibility of clinical isolates against OlPC, miltefosine, amphotericin B, and meglumine antimoniate (EC_50_ and EC_90_ in µg Sb^V^/mL) estimated from four-parameter logistic modeling.

Drug	*Leishmania* species	EC_50_ (95% CI)**	EC_90_**	Hill slope	*R* ^2^
Meglumine antimoniate	*L. major*	10.2 (8.5–11.8)	478	0.87	95.1
	*L. tropica*	10.5 (9.8–11.1)	222	0.90	95.6
Amphotericin B	*L. major*	3.9 (2.8–4.9)	2,532	0.53	95.7
	*L. tropica*	2.3 (1.9–2.7)	114	0.65	98.1
OlPC	*L. major*	8.1 (6.4–9.8)	818	0.55	96.5
	*L. tropica*	6.2 (5.1–7.3)	391	0.57	97.0
Miltefosine	*L. major*	13.5 (10.3–16.6)	8,516	0.49	94.7
	*L. tropica*	10.5 (8.4–12.6)	874	0.54	96.7

*EC_50_ (95% CI) and EC_90_ values in µg/mL**; 34 and 36 independent assays for *L. major* and *L. tropica*, respectively, and each concentration was evaluated in quadruplicate in each independent assay.

**Drug concentrations are expressed in μg/mL to facilitate direct comparison across compounds, particularly for meglumine antimoniate (μg of SbV/mL), which lacks a well-defined molecular weight due to its non-stoichiometric, polymeric nature. For consistency, this unit was maintained across all the drugs that were tested (miltefosine, OlPC, and amphotericin B). Supplementary Table S1 for EC_50_ and EC_90_ in µM for miltefosine, OlPC, and amphotericin B.

EC_50_ values were compared between OlPC and the three other drugs in both *Leishmania* species by analysis of variance (ANOVA). The results (Table 2) indicated a significant effect of the drug type on EC_50_ estimates compared to OlPC (*p* < 0.001 for each of the three drugs). Amphotericin B was associated with a significantly lower EC_50_ (difference estimate: −4.1 μg/mL, *p* < 0.001), indicating that it required lower concentrations to achieve 50% inhibition than OlPC. Meglumine antimoniate (reported as Sb^V^) had a higher EC_50_ value (difference estimate: 3.2 μg/mL, *p* < 0.001), indicating weaker potency. Most importantly, miltefosine, a close analog of OlPC currently used to treat CL, showed an elevated EC_50_ value (difference estimate: 4.8 μg/mL, *p* < 0.001), although the increase was less pronounced than that observed for meglumine antimoniate. This suggests that higher concentrations of miltefosine are required to achieve 50% inhibition of the respective *Leishmania* parasites compared with OlPC.

**TABLE 2:** Comparisons of EC_50_ values in clinical isolates between amphotericin B, meglumine antimoniate, and miltefosine, relative to OlPC.

Drug	Difference in EC_50_ (µg/mL) relative to OlPC (95% confidence interval) and *p*-value
Miltefosine	4.8 (3.5, 6.2) <0.001
Sb^V^	3.2 (1.8, 4.5) <0.001
Amphotericin B	−4.1 (−5.4, −2.7) <0.001

Although drug type significantly affected EC_50_ values, the difference in EC_50_ values between the *L. major* and *L. tropica* species was borderline in terms of statistical significance (fixed effect difference of −1.54 μg/mL, 95% CI: −3.08–−0.01, *p* = 0.054). This indicates that species susceptibility did not significantly influence drug potency in this experimental setup.

Cytotoxicity assays in THP-1 macrophages and primary fibroblasts and NHDF showed showed OlPC and miltefosine exhibited moderate toxicity in host cells, with CC_50_ values of 20.04 µM (THP-1) and 115.8 µM (NHDF) for OlPC and 27.35 µM (THP-1) and 92.05 µM (NHDF) for miltefosine. These values result in a selectivity index for OlPC that is higher in both THP-1 cells (1.2 vs 0.93) and NHDF (7.0 vs 3.1) than that for miltefosine, indicating a wider therapeutic window (Table 3).

**TABLE 3:** Cytotoxicity, anti-leishmanial activity, and selectivity index of OlPC and miltefosine.

Drug	Average EC_50_ (µM) *Leishmania*	CC_50_ (µM) THP-1 macrophages	CC_50_ (µM) NHDF	SI (THP-1)	SI (NHDF)
Miltefosine	29.45	20.0	115.8	0.9	3.1
OlPC	16.5	27.4	92.1	1.2	7.0

## Discussion

CL is caused by over 15 species of *Leishmania* (Auwera and Dujardin, 2015), each capable of producing a wide range of clinical manifestations. This inherent diversity in biochemistry, host–parasite interactions, and, importantly, drug susceptibility (Yardley et al., 2005) contributes to the diverse clinical outcomes observed and highlights the importance of selecting a well-defined and representative panel of *Leishmania* species during drug discovery to ensure that new treatments can effectively address the full spectrum of potential CL presentations (Caridha et al., 2019). Although OlPC has been tested across seven different species using our laboratory-adapted strains, in this study, we report its efficacy only against two Old World species (Van Bocxlaer et al., 2023). Further studies are needed to assess the performance of OlPC and other compounds across a wider range of clinical isolates representing the global diversity of CL-causing parasites.

We observed a higher EC_50_ for miltefosine (13.5 and 10.5 μg/mL) than for OlPC (8.1 and 6.2 μg/mL) against the *L. major* and *L. tropica* clinical isolates. This suggests that OlPC exhibits superior anti-leishmanial activity compared to miltefosine (difference in EC_50_ value of 4 μg/mL, Table 2), which remains the only oral drug available for the treatment of CL. Earlier studies using laboratory strains reported no significant difference in EC_50_ values between miltefosine and OlPC (Van Bocxlaer et al., 2023). These differences may be explained by limitations such as a small sample size (n = 2 per species), the use of long-term cultured strains, and differences in methodology. Notably, previous work assessed drug efficacy based on the percentage of infected macrophages, while our study quantified amastigote burden, which provides a more direct measure of intracellular parasite replication. Furthermore, *Leishmania* strains maintained *in vitro* are prone to phenotypic changes over time, including reduced infectivity (Piel et al., 2022)—a phenomenon mitigated by regular passage through mice to preserve virulence and ensure the reliability of experimental results. Surprisingly, the EC_50_ values for *L. major* and *L. tropica* exhibited no significant differences in susceptibility in our study, despite earlier reports suggesting that *L. tropica* may be more susceptible to miltefosine (Escobar et al., 2002). OlPC also demonstrated low cytotoxicity in both THP-1 macrophages and primary human fibroblasts, resulting in higher selectivity indices than those of miltefosine and highlighting a favorable safety profile in relevant host cell types. Amphotericin B and meglumine antimoniate (the standard treatment in Iran) were also evaluated for activity against clinical isolates. Amphotericin B emerged as the most potent, with EC_50_ values approximately 4 μg/mL lower than those of OlPC, while meglumine antimoniate (as a source of Sb^V^) showed values approximately 3 µg of Sb^V^/mL higher. These findings are consistent with previously reported data (Escobar et al., 2002; Neal and Allen, 1988).

Although the dose-response data provided useful insights into the relative potency of the drugs tested, further evaluation of our four-parameter logistic model for dose-response fitting revealed that for certain clinical isolates, the inhibition did not always plateau with higher drug concentrations. Although incomplete inhibition is not uncommon in drug testing, this may affect the accuracy of, for example, the EC_90_ values derived from the model. To analyze these data effectively, we chose R for our data analysis, which offered several key advantages over proprietary software programs, such as GraphPad Prism. R, particularly with the drc package (Ritz et al., 2016; Ritz and Streibig, 2005), provides greater flexibility and control over point-and-click software (Ritz et al., 2016), especially in efficiently handling larger datasets. It also facilitated model comparison (e.g., between models with different numbers of parameters fitted to the same data) and enabled a more rigorous assessment of goodness of fit. Using a summary measures approach (Matthews et al., 1990), we employed a linear mixed-effects model to investigate the effects of drugs and species on EC_50_, taking account of the multiple measurements within the same isolate. Although R requires a steeper learning curve than point-and-click software, its advantages in flexibility, reproducibility, and cost-effectiveness, particularly for large-scale analyses such as this one, outweigh the initial investment in learning.

In conclusion, this study highlights the superior *in vitro* anti-leishmanial activity of OlPC compared to that of miltefosine, the only currently available oral treatment for CL, and meglumine antimoniate as the standard treatment of care in Iran. By utilizing *ex vivo* clinical isolates rather than laboratory-adapted strains, our findings provide a more representative assessment of drug susceptibility in real-world settings. Our results underscore the need for further investigation into OlPC as a promising alternative to miltefosine, with the potential to improve treatment outcomes for CL.

## Data Availability

The raw data supporting the conclusions of this article will be made available by the authors, without undue reservation.
