# Supplementary material for: Comparative in vitro susceptibility of clinical Leishmania isolates to miltefosine and oleylphosphocholine
Source: Front Pharmacol. 2025 Oct 27;16:1688856. doi: 10.3389/fphar.2025.1688856 (PMC12598013; doi:10.3389/fphar.2025.1688856)
Supplement: Supplementary file 1 [file Supplementaryfile1.pdf]

Supplementary Material

Table S1. The susceptibility of clinical isolates against OlPC, miltefosine, amphotericin B (EC_{50} and EC_{90} in µM) and meglumine antimoniate (EC_{50} and EC_{90} in µg/mL) estimated from four-parameter logistic modelling.

**Drug**	***Leishmania* species**	**EC_{50} (95%CI)**	**EC_{90}**	**Hill slope**	***R^2***
**Sb^V** (µg of Sb^V/mL)	*L. major*	10.2 (8.5-11.8)	478	0.87	95.1
	L. tropica	10.5 (9.8-11.1)	222	0.90	95.6
**Amphotericin B** (µM)	*L. major*	4.2 (3.0-5.3)	2740	0.53	95.7
	L. tropica	2.5 (2.1-2.9)	123	0.65	98.1
**OlPC** (µM)	*L. major*	18.7 (14.8-22.6)	1886	0.55	96.5
	L. tropica	14.3 (11.8-16.8)	902	0.57	97.0
**Miltefosine** (µM)	*L. major*	33.1 (25.3-40.7)	20893	0.49	94.7
	L. tropica	25.8 (20.6-30.9)	2144	0.54	96.7

34 and 36 independent assays for *L. major* and *L. tropica*, respectively and each concentration was evaluated in quadruplicate in each independent assay.

Supplementary material - R code

```
#### Libraries ####

library(ggplot2)

library(dplyr)

library(drc)

library(tidyverse)

library(wesanderson)


#### Set Working Directory ####

setwd("xxxxadapt_to_your_computer_settingsxxxx")


#### Load Data ####

wideDF <- read.csv("all isolates_API_conc.csv", header = TRUE)


#### Clean Data ####

inhibition_cols <- grep("X.inhibition", names(wideDF))

wideDF[inhibition_cols] <- lapply(wideDF[inhibition_cols], function(x) ifelse(x < 0, 0, x))

wideDF <- wideDF[,-grep("ID", names(wideDF), invert = FALSE)]
```

```
#### Reshape Data ####
longDF <- reshape(data = wideDF,
         varying = grep("X.inhibition", names(wideDF)),
         sep= "",
         timevar= "ID",         direction = "long")

#### Species Assignment ####
longDF$species <- ifelse(longDF$ID <= 34, "L. major", "L. tropica")
table(longDF$ID, longDF$Drug) #each ID has only 1 species

#### Summary Statistics ####
summary_stats <- longDF %>%
  group_by(Drug, Concentration..ug.mL., species) %>%
  summarise(
    mean_inhibition = mean(X.inhibition, na.rm = TRUE),
    sd_inhibition = sd(X.inhibition, na.rm = TRUE),
    .groups = 'drop'
  )

summary_stats_intermediate <- longDF %>%
  group_by(Drug, Concentration..ug.mL., species)
summary_stats <- summary_stats_intermediate %>%
  dplyr::summarize(
    mean_inhibition = mean(`X.inhibition`, na.rm = TRUE),
    sd_inhibition = sd(`X.inhibition`, na.rm = TRUE),
    min_inhibition = min(`X.inhibition`, na.rm = TRUE),
    max_inhibition = max(`X.inhibition`, na.rm = TRUE),
    n = n()
  )

print(summary_stats)
```

```
#### Plot Inhibition Curves ####
ggplot(
  summary_stats %>% filter(!is.na(mean_inhibition) & !is.na(sd_inhibition)),  # Filter out NAs
  aes(
    x = Concentration..ug.mL.,
    y = mean_inhibition,
    color = species,
    group = interaction(Drug, species)
  )
) +
  geom_line() +
  geom_ribbon(
    aes(ymin = mean_inhibition - sd_inhibition, ymax = mean_inhibition + sd_inhibition, fill = Drug),
    alpha = 0.2
  ) +
  geom_point() +
  labs(x = "Concentration (log scale)", y = "Mean Inhibition") +
  theme_minimal() +
  scale_color_brewer(palette = "Dark2") +
  scale_x_log10(
    labels = scales::label_number(accuracy=0.1),
    expand=c(0,0)
  ) +
  facet_wrap(~ Drug, scales = "fixed")


#### 4PL Model Fitting Function ####
Fit_and_predict <- function(longDF, verbose=F, FixUpper100=F, FixLower0=F,
                            bStart=NULL, eStart=NULL) {
  # Ensure that 'Drug' is a factor
  longDF$Drug <- as.factor(longDF$Drug)
```

```
  # Initialize an empty data frame to store results
  final_result <- data.frame()
  EC_values <- data.frame(Drug = character(), EC50 = numeric(), EC90 = numeric(),
                          HillSlope = numeric(), R2 = numeric(),
                          LowerAsymptote=numeric(),
                          stringsAsFactors = FALSE)
  CI_values <- data.frame(Drug = character(), LowerCI = numeric(), UpperCI = numeric(), stringsAsFactors = FALSE)

  if(FixUpper100){
    if(FixLower0){
      coef_valuesDF<-data.frame(
        b=numeric(), e=numeric())
    }else{
      # only upper asymptote is fixed
      coef_valuesDF<-data.frame(
        b=numeric(),
        c=numeric(),
        e=numeric())
    }
  }else{
    if(FixLower0){
      # only lower asymptote is  fixed, at 0
      coef_valuesDF<-data.frame(
        b=numeric(),
        d=numeric(),
        e=numeric())
    }else{
      # neither  asymptote is fixed
      coef_valuesDF<-data.frame(
        b=numeric(),
        c=numeric(),
        d=numeric(),
```

```
    e=numeric())
  }
}
coef_valuesDF$Drug=character()
if(verbose){
  print("coef_valuesDF:")
  print( coef_valuesDF  )
}

# Loop through each drug and fit the model separately
for (drug in unique(longDF$Drug)) {
  cat("###\n", "Processing drug:", drug, "\n###\n")

  # Subset the data for the current drug
  drug_data <- subset(longDF, Drug == drug)
  drug_data <- drug_data[!is.na(drug_data$X.inhibition) & !is.na(drug_data$Concentration..ug.mL.),]

  # if drug is Amphotericin, set initial values
  if(!is.null(bStart) & !is.null(eStart)){
    AmphotericinInits<-unlist(list(b=bStart, e=eStart))
    if(verbose){
      print("AmphotericinInits:")
      print(AmphotericinInits)
    }
  }

  # Fit the model
  if(FixUpper100){
    if(FixLower0 | drug=="Miltefosine" | drug=="OlPC"){ # impose  FixLower0==T for certain drugs
      # both asymptotes are fixed
      if(drug=="Amphotericin B" | drug=="Glucantime"){
        coef_valuesDF<-data.frame(
```

```
      b=numeric(), e=numeric())}
    args <- list(
      X.inhibition ~ Concentration..ug.mL.,
      fct = LL.4(fixed = c(NA, 0, 100, NA), names = c("b", "c", "d", "e")),
      data = drug_data)
    if((drug=="Amphotericin B") & !is.null(bStart) & !is.null(eStart)) {
      args$start <- c(AmphotericinInits["b"], AmphotericinInits["e"])
    }
    model <- try(do.call(drm, args), silent = TRUE)
    # model <- try(
    #   drm(X.inhibition ~ Concentration..ug.mL., data = drug_data,
    #       fct =LL.4(fixed = c(NA, 0, 100, NA), names = c("b", "c", "d", "e")),
    #   ),
    #   silent = TRUE)
  }else{
    # only upper asymptote is fixed
    model <- try(
      drm(X.inhibition ~ Concentration..ug.mL., data = drug_data,
          fct =LL.4(fixed = c(NA, NA, 100, NA), names = c("b", "c", "d", "e"))),
      silent = TRUE)
  }
}else{
  if(FixLower0){
    # only lower asymptote is  fixed, at 0
    model <- try(
      drm(X.inhibition ~ Concentration..ug.mL., data = drug_data,
          fct =LL.4(fixed = c(NA, 0, NA, NA), names = c("b", "c", "d", "e"))),
      silent = TRUE)
  }else{
    # neither  asymptote is fixed
    model <- try(drm(X.inhibition ~ Concentration..ug.mL., data = drug_data,
                 fct =LL.4(fixed = c(NA, NA, NA, NA), names = c("b", "c", "d", "e"))),
```

```
                silent = TRUE)
  }
}

if (inherits(model, "try-error")) {
  cat("Model fitting failed for drug:", drug, "\n")
  next
} else {
  if(verbose){(cat("Model fitting succeeded for drug:", drug, "\n"))}

  # 1. Calculate EC50 and EC90
  ec50 <- try(invisible(ED(model, 50)), silent = TRUE)
  ec90 <- try(invisible(ED(model, 90)), silent = TRUE)

  if (!inherits(ec50, "try-error") && !inherits(ec90, "try-error")) {
    if(verbose){
      cat("EC50 for drug", drug, ":", ec50, "\n")
      cat("EC90 for drug", drug, ":", ec90, "\n")
    }
  } else {
    if(verbose){
      cat("EC50 or EC90 calculation failed for drug:", drug, "\n")
    }
    next
  }

  # 2. Extract Hill slope
  if(verbose){
    print("coef(model):")
    print( coef(model))
  }
  # hill_slope <- coef(model)[3]  # Third parameter is the Hill slope
```

```
hill_slope <- -1*coef(model)["b:(Intercept)"]  # this parameter is the Hill slope

# 3. Calculate R²
predictions <- predict(model)
if(length(drug_data$X.inhibition)!=length(predictions)){
  print(paste0("Warning: lengths of X.inhibition is ",
               length(drug_data$X.inhibition),
               ", but length of predictions is ",
               length(predictions)))
}
rss <- sum((drug_data$X.inhibition - predictions)^2)  # Residual sum of squares
tss <- sum((drug_data$X.inhibition - mean(drug_data$X.inhibition))^2)  # Total sum of squares
r_squared <- 1 - rss/tss  # R-squared

# 4. Confidence Intervals for EC50
ci <- try(confint(model, level = 0.95), silent = TRUE)  # 95% CI
if (!inherits(ci, "try-error")) {
  lower_ci <- ci["e:(Intercept)", 1]  # Lower CI for EC50
  upper_ci <- ci["e:(Intercept)", 2]  # Upper CI for EC50
  if(verbose){
    cat("95% CI for EC50 for drug", drug, ":", lower_ci, "-", upper_ci, "\n")
  }
} else {
  lower_ci <- NA
  upper_ci <- NA
  if(verbose){
    cat("Confidence interval calculation failed for drug:", drug, "\n")
  }
}

# extract lower asymptote
if("c:(Intercept)" %in% names(coef(model))){
```

```
  LowerAsymptoteScalar=coef(model)["c:(Intercept)"]
}else{
  LowerAsymptoteScalar<-0
}

# Store EC values and CIs
EC_values <- rbind(EC_values,
                   data.frame(
                     Drug = drug, EC50 = ec50, EC90 = ec90,
                     HillSlope = hill_slope, R2 = r_squared,
                     LowerAsymptote=LowerAsymptoteScalar))
CI_values <- rbind(CI_values, data.frame(Drug = drug, LowerCI = lower_ci, UpperCI = upper_ci))
if(verbose){
  print("coef_valuesDF before rbind:")
  print( coef_valuesDF  )
}
NameVector<-names(coef_valuesDF) # this is the cumulative DF of values
if(verbose){
  print("NameVector:")
  print( NameVector)
}
coef_model<-as.data.frame(t(coef(model)))
if(verbose){
  print("coef_model:")
  print( coef_model)
}
coef_model$Drug<-""
if(verbose){
  print("coef_model as data frame, having initialized the Drug field:")
  print( coef_model  )
}
if(! "c:(Intercept)" %in% names(coef_model) & "c" %in% names(coef_valuesDF)){
```

```
  # add a column to coef_model (which is a data frame)
  coef_model[["c:(Intercept)"]]<-NA
  coef_model <- coef_model[, c(1, ncol(coef_model), 2:(ncol(coef_model) - 1))]
}
names(coef_model)[1:length(NameVector)]<-NameVector
coef_model$Drug<-drug
if(verbose){
  print("coef_model after renaming:")
  print( coef_model  )
}
coef_valuesDF<-rbind(coef_valuesDF, coef_model)
if(verbose){
  print("coef_valuesDF after rbind:")
  print( coef_valuesDF  )
}

# Generate concentration sequence for predictions
conc <- 10**seq(
  log10(min(drug_data$Concentration..ug.mL., na.rm = TRUE)),
  log10(max(drug_data$Concentration..ug.mL., na.rm = TRUE)),
  length.out = 100)

# Predict inhibition values
pred <- predict(model, newdata = data.frame(Concentration..ug.mL. = conc))

# Check prediction result length
if (length(pred) != length(conc)) {
  if(verbose){
    cat("Prediction length mismatch for drug:", drug, "\n")
  }
  next
}
```

```
    # Create result data frame for predictions
    result <- data.frame(
      Drug = rep(drug, length(conc)),
      Concentration = conc,
      X.inhibition = pred
    )

    # Append to final result
    final_result <- rbind(final_result, result)
  }
 }

 # Return results: predictions, EC values, and CIs
 if (nrow(final_result) == 0) {
  if(verbose){
    cat("No results generated.\n")
  }
 } else {
  if(verbose){
    cat("Results successfully generated.\n")
  }
 }

 return(list(predictions = final_result, EC_values = EC_values, CI_values = CI_values, coef_values=coef_valuesDF))
}

print( summary(longDF))

##### Fit Models and Save Results ####
# Call the function and capture results in a list
results <- Fit_and_predict(longDF)
```

```
print(results$coef_values)#b=slope, c=bottom response, d=top response and e=EC50

# look at the predictions
print(   head(results$predictions))
print(summary(results$predictions))

#Fit by species
resultsMajor          <-Fit_and_predict(longDF[longDF$species=="L. major", ], verbose=FALSE)
PredictionsMajor      <-resultsMajor$predictions
PredictionsMajor$species  <-"L. major"

resultsTropica        <-Fit_and_predict(longDF[longDF$species=="L. tropica", ], verbose=FALSE)
PredictionsTropica    <-resultsTropica$predictions
PredictionsTropica$species<-"L. tropica"

PredictionsSpecies<-rbind(PredictionsMajor, PredictionsTropica)

# make a data frame with both the predictions and the data
names(summary_stats)[names(summary_stats)   == "Concentration..ug.mL."] <- "Concentration"
summary_stats$predict.inhibition<-NA
summary_stats <- summary_stats[, setdiff(
  names(summary_stats),
  c("n", "min_inhibition", "max_inhibition"))]
print(names(summary_stats))

names(PredictionsSpecies)[names(PredictionsSpecies)   == "X.inhibition"] <- "predict.inhibition"
PredictionsSpecies$mean_inhibition <- NA
PredictionsSpecies$sd_inhibition   <- NA
print(names(PredictionsSpecies))

table(sort(names(PredictionsSpecies))==sort(names(summary_stats)))
```

```
PredictionsSpecies <- PredictionsSpecies[,
                    match(names(summary_stats), names(PredictionsSpecies))]

DrugPlot<-ggplot() +
  geom_ribbon(data=summary_stats, aes(x=Concentration, ymin = mean_inhibition - sd_inhibition, ymax = mean_inhibition + sd_inhibition, fill = Drug, group = interaction(Drug, species)), alpha = 0.2) +
  geom_line(data=PredictionsSpecies, aes(x = Concentration, y = predict.inhibition, color = species, group = interaction(Drug, species))) +
  labs(x = "Concentration (log scale)", y = "Mean Inhibition") +
  theme_minimal() +
  scale_color_brewer(palette = "Dark2") +
  scale_x_log10(
    breaks = scales::trans_breaks("log10", function(x) 10^x),
    labels = scales::trans_format("log10", scales::math_format(10^.x))
  ) +
  facet_wrap(~ Drug, scales = "free")

print(DrugPlot)

#### make a wrapper function to extract only predictions ####
PredictOnly<-function(longDF, ...){
  Fit_and_predict(longDF=longDF, ...)$predictions
}

# Call the function and capture results in a list
# run it on one person's data - individual level fits
head(table(longDF$ID))

resultsID1 <- Fit_and_predict(longDF[longDF$ID==1,], verbose=T)
EC_ID1<-resultsID1$EC_values
head(EC_ID1)
EC_ID1$ID<-1
```

```
Predictions_ID1<-resultsID1$predictions
Predictions_ID1$ID<-1

# use this to make DFs with no rows
ParameterDF   <- EC_ID1[0, ]
PredictionsDF <- Predictions_ID1[0, ]

# omit missing values
dim(longDF)
longDF<-longDF[!is.na(longDF$X.inhibition),]
dim(longDF)

#check poor fit curves
StartDF<-data.frame(
  ID=  c(20, 22, 26, 42),
  b=rep(0.76, 4),
  e=  c(5, 10, 10, 7))
StartDF

IDVector<-sort(unique(longDF$ID))
for(i in 1:length(IDVector)){
  # for(i in 1:10){
  IDScalar          <-IDVector[i]
  print("##########")
  print(paste0("ID=", IDScalar))
  print("##########")

  # resultsSubset     <-Fit_and_predict(longDF[longDF$ID==IDScalar,], verbose=F,
  #                                     FixUpper100=T, FixLower0=T)
  if(IDScalar %in% StartDF$ID){
    bStartScalar<-StartDF[StartDF$ID==IDScalar, "b"]
    eStartScalar<-StartDF[StartDF$ID==IDScalar, "e"]
```

```
  }else{
   bStartScalar<-eStartScalar<-NULL
  }
  resultsSubset     <-Fit_and_predict(longDF[longDF$ID==IDScalar,],
                                     FixUpper100=T,
                                     FixLower0=!(IDScalar %in% c(9)),
                                     bStart=unname(bStartScalar), eStart=unname(eStartScalar),
                                     verbose=I(IDScalar==9))
  # FixLower0=I(IDScalar!=9))
  PredictionsSubset   <-resultsSubset$predictions
  PredictionsSubset$ID<-IDScalar
  PredictionsDF       <-rbind(PredictionsDF, PredictionsSubset)

  ParameterSubset   <-resultsSubset$EC_values
  ParameterSubset$ID<-IDScalar
  ParameterDF       <-rbind(ParameterDF, ParameterSubset)
}
# Add species info
  ParameterDF$species <- ifelse(  ParameterDF$ID <= 34, "L. major", "L. tropica")
PredictionsDF$species <- ifelse(PredictionsDF$ID <= 34, "L. major", "L. tropica")

# Join species info into PredictionsDF
PredictionsDF <- dplyr::left_join(PredictionsDF, ParameterDF[, c("ID", "species")], by = "ID")

# function to generate 95% confidence interval for mean
ConfIntFn<-function(x, percentile=0.95){
  x      <-x[!is.na(x)]
  n      <- length(x)
  mean_x <- mean(x)
  stderr <- sd(x) / sqrt(n)
  t_value<- qt(1-((1-percentile)/2), df = n - 1)
  lower_bound <- mean_x - t_value * stderr
```

```
  upper_bound <- mean_x + t_value * stderr
  return(c(lower_bound, upper_bound))
}

DrugVector<-sort(unique(ParameterDF$Drug))
print(DrugVector)
DrugVector<-DrugVector[c(3,2,1,4)]
print(DrugVector)

table(ParameterDF$Drug, ParameterDF$species)

#Create results table and save in working directory folder
WriteTable<-function(DF, filename="Table1.txt", verbose=F){
  SpeciesVector <-c("L. major", "L. tropica")

  # Write header line first (overwrite file)
  header <- paste(
    sprintf("%-12s", "Drug"),
    sprintf("%-10s", "Species"),
    sprintf("%-18s", "EC50"),
    sprintf("%-8s", "EC90"),
    sprintf("%-7s", "Slope"),
    sprintf("%-6s", "R²"),
    sep = " | "
  )
  cat(header, "\n", file = filename)

  for (drugScalar in DrugVector) {
    for (speciesScalar in SpeciesVector) {

      if (verbose) print(unlist(list(Drug = drugScalar, Species = speciesScalar)))
```

```
      subsetVector <- DF$Drug == drugScalar & DF$species == speciesScalar
      DFSubset <- DF[subsetVector, ]

      if (nrow(DFSubset) == 0) {
        cat(paste(drugScalar, speciesScalar, "-> No data available\n"))
        next
      }

      EC50mean <- mean(DFSubset$EC50.Estimate, na.rm = TRUE)
      EC50ConfInt <- ConfIntFn(DFSubset$EC50.Estimate)
      EC50Lo <- EC50ConfInt[1]
      EC50Hi <- EC50ConfInt[2]
      EC90mean <- mean(DFSubset$EC90.Estimate, na.rm = TRUE)
      HillSlopeMean <- mean(DFSubset$HillSlope, na.rm = TRUE)
      R2mean <- mean(DFSubset$R2, na.rm = TRUE)
      RowString <- paste(
        sprintf("%-12s", drugScalar),
        sprintf("%-10s", speciesScalar),
        sprintf("EC50: %.1f (%.1f–%.1f)", EC50mean, EC50Lo, EC50Hi),
        sprintf("%.0f", EC90mean),
        sprintf("%.2f", HillSlopeMean),
        sprintf("%.1f%%", 100 * R2mean),
        sep = " | "
      )

      cat(RowString, "\n", file = filename, append = TRUE)
    }
  }
}

table(ParameterDF$strainType)
names(ParameterDF)
```

```
table(ParameterDF$species)
WriteTable(DF=ParameterDF, verbose=T,
        filename=paste0("Table1 ", gsub("-", " ", Sys.Date()), ".txt")
        )

####Compare EC50's to those of OlPC####
LL.4PredictFn<-function(x, b, c, d, e){
  c + (d - c) / (1 + exp(b * (log(x) - log(e))))
}

MeanPredictDF<-data.frame(
  Drug=character(), Concentration=numeric(0), X.inhibition=numeric(0))

for(i in 1:length(DrugVector)){
  DrugScalar=DrugVector[i]
  # extract mean EC50 and slope from ParameterDF
  SubsetVector<-ParameterDF$ID<=70 & ParameterDF$Drug==DrugScalar
  ConcPredictVector<-sort(unique(PredictionsDF[
    PredictionsDF$Drug==DrugScalar & PredictionsDF$ID<=70, "Concentration"]))
  EC50mean     <-mean(ParameterDF[SubsetVector, "EC50.Estimate"])
  HillSlopemean<-mean(ParameterDF[SubsetVector, "HillSlope"])
  InhibitPredictVector<-LL.4PredictFn(x=ConcPredictVector,
                              b=-1*HillSlopemean, c=0, d=1, e=EC50mean)
  MeanPredictDrugDF<-data.frame(
    Drug=rep(DrugScalar, length(ConcPredictVector)),
    Concentration=ConcPredictVector,
    X.inhibition=100*InhibitPredictVector)
  # plot(x=log10(ConcPredictVector), y=InhibitPredictVector, type="l", main=DrugScalar)
  MeanPredictDF<-rbind(MeanPredictDF, MeanPredictDrugDF)
}
table(MeanPredictDF$Drug)
```

```
summary(MeanPredictDF)

# 2025 07 16 the following was not working because of "OlPC" vs "OlPC"
# fix by using "toupper"
plot(x=log10(MeanPredictDF[toupper(MeanPredictDF$Drug)=="OLPC", "Concentration"]),
    y= MeanPredictDF[toupper(MeanPredictDF$Drug)=="OLPC", "X.inhibition"], type="l",
main=DrugScalar)

#summary plot predictions-means only
ggplot(MeanPredictDF,
       aes(x = Concentration, y = X.inhibition, color = Drug)) +
  geom_line(aes(group = Drug)) +
  scale_x_log10() +
  theme_minimal() +
  labs(x = "Concentration (log scale)",
       y = "Percent Inhibition",
       title = "Inhibition vs. Concentration by Drug and ID")

#summary plot predictions-all
ggplot(PredictionsDF[PredictionsDF$ID<=70,],
       aes(x = Concentration, y = X.inhibition, color = Drug)) +
  geom_line(aes(group = interaction(ID, Drug)), alpha = 0.1, linewidth=0.75) +
  geom_line(data = MeanPredictDF, linewidth = 1.2) +
  geom_hline(yintercept = 50, color = "black", linewidth = 0.25, linetype = "dashed") +
  scale_x_log10() +
  theme_minimal() +
  labs(x = "Concentration (µg/mL, log scale)",
       y = "Percent Inhibition")


IndividualPlotFn<-function(IDScalar, fitDF=PredictionsDF, dataDF=longDF,
                           SpeciesDF=SpeciesByID, verbose=F){
  # SpeciesScalar<-ifelse(
```

```
#   SpeciesDF[SpeciesDF$ID==IDScalar,"major"], "major", "tropica")
SpeciesScalar<-SpeciesDF[SpeciesDF$ID==IDScalar,"species"]
# harmonize names between the data frames
names(dataDF)[names(dataDF)   == "Concentration..ug.mL."] <- "Concentration"
# subset on variables and on ID
dataDF<-dataDF[
  dataDF$ID==IDScalar,
  c("Concentration", "X.inhibition", "Drug")]
dataDF$X.inhibition.fit<-NA
# distinguish fits from data in terms of variable name
names(fitDF)[names(fitDF) == "X.inhibition"]         <- "X.inhibition.fit"
# subset on variables and on ID
fitDF<-fitDF[
  fitDF$ID==IDScalar,
  c("Concentration", "X.inhibition.fit", "Drug")]
fitDF$X.inhibition<-NA
OrderVector <-colnames(dataDF)
fitDF     <-fitDF[,OrderVector]
DataWithFitsDF<-rbind(dataDF, fitDF)
# print( dim(DataWithFitsDF))
# print(head(DataWithFitsDF))
if(verbose){
  print("Summary of concentration for rows with non-missing fitted value of inhibition:")
  print(aggregate(Concentration ~ Drug,
                data = DataWithFitsDF[!is.na(DataWithFitsDF$X.inhibition.fit),], summary))}
if(verbose){
  print("Summary of fitted inhibition:")
  print(aggregate(X.inhibition.fit ~ Drug,
                data = DataWithFitsDF[!is.na(DataWithFitsDF$X.inhibition.fit),], summary))}
if(verbose){
  print("Summary of observed inhibition:")
  print(aggregate(X.inhibition ~ Drug,
```

```
                data = DataWithFitsDF[!is.na(DataWithFitsDF$X.inhibition),], summary))}

  print(ggplot(DataWithFitsDF, aes(x = Concentration, color = Drug)) +
        # Add lines for each drug using Xfit
        geom_line(aes(y = X.inhibition.fit)) +
        # Add points for each drug using Xdata
        geom_point(aes(y = X.inhibition)) +
        # Customize the plot as needed
        labs(x = "Concentration", y = "Response") +
        ggtitle(paste0("ID=", IDScalar, ", species=", SpeciesScalar)) +
        scale_x_log10() +
        theme_minimal())

}

head(ParameterDF)

SpeciesVector<-c("L. major", "L. tropica")

table(ParameterDF$species)

summary(ParameterDF)

hist(log10(ParameterDF$HillSlope))
summary(ParameterDF$HillSlope)

tapply(
  ParameterDF$HillSlope,
  list(ParameterDF$species, ParameterDF$Drug),
  mean)

vars_to_log <- c("EC50.Estimate", "HillSlope")
```

```r
stacked <- stack(ParameterDF[vars_to_log])
long_df <- data.frame(
  Drug     = rep(ParameterDF$Drug, times = length(vars_to_log)),
  species = rep(ParameterDF$species, times = length(vars_to_log)),
  Variable = stacked$ind,
  Value    = stacked$values
)
long_df$LogValue <- log10(long_df$Value)

for (j in 1:length(SpeciesVector)) {
  df_sub <- subset(long_df, species == SpeciesVector[j])
  p <- ggplot(df_sub, aes(x = LogValue)) +
    geom_histogram(binwidth = 0.1, color = "black", fill = "steelblue") +
    facet_grid(Variable ~ Drug, scales = "fixed") +
    labs(title = paste("Species:", SpeciesVector[j]), x = expression(log[10]~value), y = "Count") +
    theme_minimal()
  print(p)
}

ParameterDF[
  ParameterDF$species=="L. major" &
  ParameterDF$Drug   =="Miltefosine" &
  log10(ParameterDF$EC50.Estimate)<I(-1),
]

# IndividualPlotFn(ID=9, verbose=F)

CheckDF<-ParameterDF[
  ParameterDF$species=="L. major" &
  ParameterDF$Drug   =="Amphotericin B" &
  log10(ParameterDF$HillSlope)>1,
  c("Drug", "EC50.Estimate", "HillSlope", "ID", "species")
```

```
]

SpeciesByID <- aggregate(species ~ ID, data = longDF, FUN = function(x) x[1])
dim(SpeciesByID)
head(SpeciesByID)

IDVector<-CheckDF$ID
IDVector
if(length(IDVector)>0){
  for(i in 1:length(IDVector)){
    IndividualPlotFn(ID=IDVector[i], verbose=F)
  }
}

####histogram bars####

# table(ParameterDF$strainType)
hist(log10(ParameterDF[ParameterDF$species=="L. major","EC50.Estimate"]))

# Ensure Drug column exists and is character
ParameterDF$Drug <- as.character(ParameterDF$Drug)
ParameterDF$Drug <- trimws(ParameterDF$Drug)

# Ensure the required columns exist before subsetting
required_cols <- c("ID", "species", "EC50.Estimate")
missing_cols <- setdiff(required_cols, names(ParameterDF))

if (length(missing_cols) > 0) {
  stop(paste("Missing columns in ParameterDF:", paste(missing_cols, collapse = ", ")))
}

# Subset OlPC control group
```

```
controlDF <- ParameterDF[ParameterDF$Drug == "OlPC", c("ID", "species", "EC50.Estimate")]
colnames(controlDF)[3] <- "EC50.OlPC"

# Drop duplicates if any
controlDF <- unique(controlDF)

# Subset non-OLPC
nonControlDF <- ParameterDF[ParameterDF$Drug != "OlPC", ]

# Merge on ID and major
diffDF <- merge(nonControlDF, controlDF, by = c("ID", "species"))

# Calculate EC50 difference
diffDF$EC50.diff <- diffDF$EC50.Estimate - diffDF$EC50.OlPC

# Select and print
diffDF <- diffDF[, c("ID", "Drug", "species", "EC50.Estimate", "EC50.OlPC", "EC50.diff")]
print(head(diffDF))

ggplot(diffDF, aes(x = EC50.diff)) +
  geom_histogram(binwidth = 5, fill = "lightblue", color = "black") +
  facet_grid(species ~ Drug) +
  theme_minimal() +
  labs(title = "EC50 Difference from OlPC by Drug and Species",
       x = "EC50 Difference",
       y = "Count")



#### lme4 ####
require(lme4) #fitting linear mixed-effects models
require(lmerTest) # provides p values and tests for lmer models
```

```
names(ParameterDF)
ParameterDF$DrugFactor<-as.factor(ParameterDF$Drug)
levels(ParameterDF$DrugFactor)
ParameterDF$DrugFactor <- relevel(ParameterDF$DrugFactor, ref = "OlPC")#olpc is set as reference

#fit linear mixed-effects model
InteractionModel <- lmer(EC50.Estimate ~ DrugFactor * as.factor(species) + (1 | ID),
                         data = ParameterDF)
summary(InteractionModel)
anova(InteractionModel)

#fit additive model
AdditiveModel    <- lmer(EC50.Estimate ~ DrugFactor + as.factor(species) + (1 | ID),
                         data = ParameterDF)
summary(AdditiveModel)
anova(AdditiveModel)

#compares two models statistically
anova(InteractionModel, AdditiveModel)

AdditiveModelconfint<-confint(AdditiveModel)
AdditiveModelconfint

# AdditiveModelcoef<-coef(summary(as(AdditiveModel,"merModLmerTest")))
AdditiveModelcoef<-coef(summary(AdditiveModel))
AdditiveModelcoef

#Prepare table with results
DrugShortVector <- DrugVector[toupper(DrugVector) != "OLPC"]
DrugShortVector

AdditiveModelconfint["DrugFactorAmphotericin B",]
```

```
AdditiveModelcoef["DrugFactorAmphotericin B",]

DecimalsScalarEstimate<-1
DecimalsScalarPValue  <-2

for(i in 1:length(DrugShortVector)){
  DrugScalar<-paste0("DrugFactor", DrugShortVector[i])
  print(DrugScalar)
  pValueScalar<-AdditiveModelcoef[DrugScalar, "Pr(>|t|)"]
  print(pValueScalar)
  pValueCharacter<-ifelse(
    pValueScalar<0.001,
    "<0.001",
    as.character(round(pValueScalar, DecimalsScalarPValue)))
  DrugScalarPrint<-paste0(DrugShortVector[i], ifelse(DrugShortVector[i]=="Amphotericin B","B",  ""))
  cat(
    paste0(
      DrugScalarPrint, "\t",
      round(AdditiveModelcoef[DrugScalar,"Estimate"], DecimalsScalarEstimate),
      " (",
      round(AdditiveModelconfint[DrugScalar, 1], DecimalsScalarEstimate),
      ",",
      round(AdditiveModelconfint[DrugScalar, 2], DecimalsScalarEstimate),
      ") ",
      pValueCharacter,
      "\n"),
    file=paste0("Table2ANOVA ", gsub("-", " ", Sys.Date()), ".txt"),
    append=I(i>1))
}
```
